# Anthropogenic particles in coypu (*Myocastor coypus*; Mammalia, Rodentia)’ faeces: first evidence and considerations about their use as track for detecting microplastic pollution

**DOI:** 10.1007/s11356-022-21032-0

**Published:** 2022-06-04

**Authors:** Luca Gallitelli, Corrado Battisti, Loris Pietrelli, Massimiliano Scalici

**Affiliations:** 1 Department of Sciences, University of Rome Tre, Rome, Italy; 2 ‘Torre Flavia’ LTER (Long Term Ecological Research) Station, Protected Areas Service, Città Metropolitana di Roma Capitale, Rome, Italy; 3 Department of Chemistry, Sapienza University of Rome, P.le A. Moro, 5, 00185 Rome, Italy

**Keywords:** Plastic pollution, Microplastics, FT-IR analysis, Wetland pollution indicator, Plastic ingestion, Alien species

## Abstract

Anthropogenic plastic litter is widespread in all environments, with particular emphasis on aquatic habitats. Specifically, although freshwater mammals are important as they are at the top of food web, research mainly focus on marine animals, while only few studies have been carried out on freshwater mammals. The main gap is that microplastics (MP) are completely understudied in freshwater mammals. Here, we reported the first evidence of the presence of anthropogenic particles (including MP) in coypu *(Myocastor coypus*)’ faeces. Coypu is a rodent mammal inhabiting rivers and wetland areas, and we discussed our preliminary data suggesting the use of these tracks as possible future bioindicator of MP pollution in wetlands and freshwaters. We collected 30 coypu’s faeces in “Torre Flavia wetland” nature reserve. Then, in laboratory, faeces were digested in 30 ml hydrogen peroxide (30%) for a week a 20 °C and analysed under stereoscope. All the suspected found MP were isolated in a petri dish, using FT-IR analysis to confirm the polymers. Overall, we recorded 444 natural and anthropogenic particles with most of items being fibres. FT-IR analysis of the 10% of the particles recovered revealed that 72% of them was not MP (mainly, polyethylene, polyethylene terephthalate, and polyamide). Also, the number of anthropogenic particles is not correlated with the faecal weight. Given that alien species, such as coypu, are widespread species, our results might have a great importance as these species and MP in faecal tracks may be used as undirect proxy of environmental bioavailability of MP pollution.

**Supplementary Information:**

The online version contains supplementary material available at 10.1007/s11356-022-21032-0.

## Introduction

Anthropogenic litter is widespread in all environments, to date. However, while marine ecosystems have been more investigated, anthropogenic litter occurring in freshwaters (i.e. rivers, lakes, and wetlands) are still poor studied (Andrady 2003; Gallitelli et al. 2020; Cera et al. 2020). Here, rivers represent lotic habitats that transport land-based plastics waste to the sea (Lebreton et al. 2017), while lakes and coastal wetlands are close ecosystems that allow plastic to sink.

Litter pollution in coastal wetlands is understudied (Cresta and Battisti 2021) and few evidence has been provided for these ecosystems (Forero-López et al. 2021; Kumar et al. 2021; Qian et al. 2021). Few information assessed the sources and origin of litter in wetlands, indicating sewage, aquaculture waste, and runoff as primary sources of these anthropogenic particles in wetlands (Qian et al. 2021). Moreover, litter distribution in wetlands may be affected by hydrodynamic, sediment, and vegetation conditions; however, in closed bodies, plastics tend to sink (Gallitelli et al. 2021b; Cresta and Battisti 2021). In addition, solar radiation and microbes have the central role in degrading and fragmenting microplastics (hereafter, MP) into nanoplastics that may enter more easily the “plastic cycle” (see Bank and Hansson 2019).

Anthropogenic litter can be distinguished using different criteria (chemical composition, size, color, origin, etc.; e.g. Kooi and Koelmans 2019). Considering size as criterion, litter can be segregated in three main categories: macro-plastic (size: > 25 mm), meso-plastics (5–25 mm), and microplastics (0.001–5 mm) (Lippiatt et al. 2013).

Regarding MP, to assess their availability in the ecosystems, bioavailability estimates are largely used verifying its presence in biological organisms that ingest or accumulate these anthropogenic materials (Fossi et al. 2018).

Although studies focused on the presence of MP ingested or accumulated in biological organisms (mainly, vertebrates) are largely available for marine ecosystems (reviews in Poeta et al. 2017; Staffieri et al. 2018; Battisti et al. 2019), analogous research carried out in freshwater and terrestrial ecosystems are still scarce (see also Blettler et al. 2018; Azevedo-Santos et al. 2021; Cera and Scalici 2021).

Among freshwater and terrestrial vertebrates, mammals represent a class of elusive vertebrates, often difficult to be seen. In this regard, their tracks (e.g. faeces) can be used for evaluating their diet, physiological fitness, and others, as for example, the ingestion of MP particles.

The analysis of mammal faeces can be useful to verify the bio-magnification, i.e. the accumulation of chemical products along the food web (O'Connor et al. 2022). Bioaccumulation and biomagnification are processes that can occur for small (1–100 μm) and large MP (> 100 μm), potentially posing a great wide ecological risk to biodiversity and ecosystem (Covernton et al. 2021). In this regard, MP recorded in faeces could be used as an undirect proxy of MP found along the trophic level (herbivorous, carnivorous, top predators). However, although the high importance of this type of tracks (Jobling 1987; Cottrell et al. 1996), few is known on MP in faeces in freshwater vertebrates (see Reynolds and Ryan 2018; in otter *Lutra lutra*, see Smiroldo et al. 2019; D’Souza et al. 2020), and data for mammals are still lacking. This is due to a large emphasis on the anthropogenic litter (and its magnification along of food webs) into the sea: in fact, research mainly focus on marine animals (see Nelms et al. 2019; Santillán et al. 2020; Zantis et al. 2021), while only few studies have been carried out on freshwater mammals (Blettler et al. 2018; Smiroldo et al. 2019; Azevedo-Santos et al. 2021), although freshwater mammals are important as they are at the top of food web.

Here, we reported the first evidence of the presence of anthropogenic particles (including MP) in coypu *(Myocastor coypus*)’ faeces. Coypu is a mammal (rodent) inhabiting wetland areas and rivers, and we discussed our preliminary data suggesting the use of these tracks as possible future bioindicator of MP pollution in wetlands and freshwaters. Indeed, a further aim was to test if this species matches the classical criteria used to select a suitable indicator of plastic occurrence in freshwater habitats.

Concerning the model species, we focused on coypu (*Myocastor coypu* Molina, 1782), an invasive semi-aquatic rodent introduced to North America as well as in several European countries as a domestic furbearer, which is now widely diffused also in Italy (Bertolino and Genovesi 2007b; Battisti et al. 2015). This species occurs mainly in plain landscapes with the presence of wet habitats. Since generally these habitat types have a patchy distribution in Mediterranean landscapes, coypu is usually spatially distributed with a meta-population structure (for details on local distribution in the study area: Battisti et al. 2015). This aquatic rodent is native to South America and, from the 1920s, was imported for farming in Europe, Asia, Africa, and North America, over the past century (Bertolino and Genovesi 2007a; Bertolino et al. 2012). This mammal repeatedly escaped from the farms or was released into the wild, and several populations have become established along riverbanks, lakes, and wetlands. This species is considered a pest because of its negative impact on biological diversity, ecological relationships, and agriculture (Kaplan et al. 1998; Carter et al. 1999; IUCN SSC Invasive Species Specialist Group 2000; Johnson-Randall and Foote 2005; Abbas 1988; Panzacchi et al. 2007; Bertolino and Viterbi 2010; Bertolino et al. 2011, 2012).

## Methods

### Study area

**Fig. 1:**
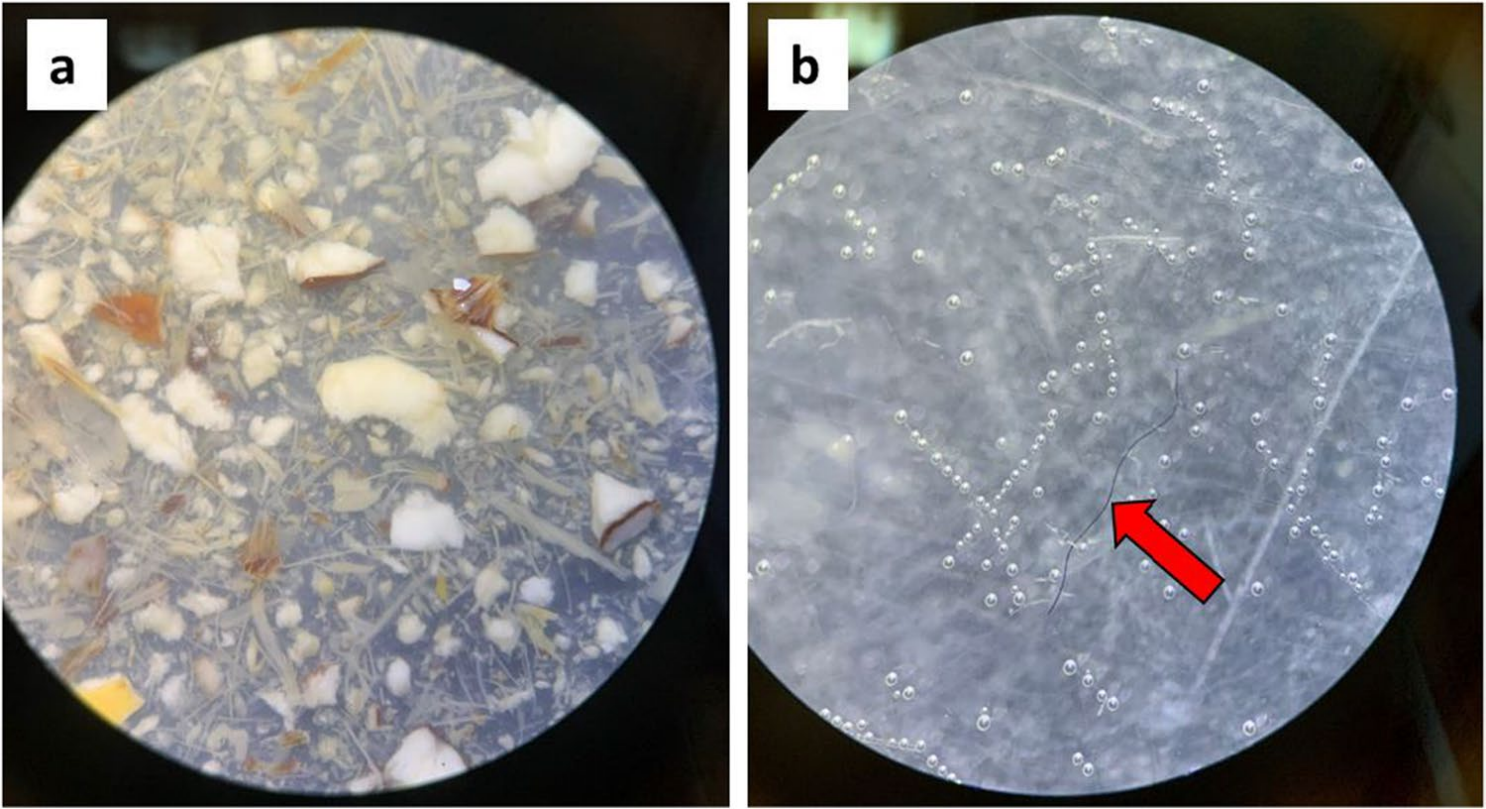
**a** Organic matter in coypu faeces and **b** a blue anthropogenic filament occurring within the coypu faeces is indicated by the red arrow.

The study area was located into the “Torre Flavia wetland” nature reserve (municipalities of Cerveteri and Ladispoli; Lazio, central Italy; 41°58′ N, 12°03′ E), a small coastal wetland (40 ha) on the Tyrrhenian coast (Special Protection Area according to the Directive 2009/147/EC “Birds”; code IT6030020), relict of a larger wetland drained and transformed by land reclamation (Battisti et al. 2006; Causarano and Battisti 2009; Fig. 1). At a local scale, this wet area shows a seminatural heterogeneity with a dominance of *Phragmites australis* reedbeds and ponds used for fish farming from 1938. From 2004, activities of fish stock management like flooding, reedbed mowing, and burning (Battisti et al. 2009a; Battisti et al. 2009b) were completely abandoned. Near the reedbeds, there are flooded meadows with *Carex hirta, Juncus acutus*, and Cyperaceae corresponding to the *Juncetalia maritimi* habitat type according to the “Habitat” Directive 92/43/EC (Guidi 2006; Fanelli and Bianco 2007). The water flooding the wetland is mainly of meteoric and sea storm origin. Climate is xeric-meso-Mediterranean (Blasi and Michetti 2005). For a faunal arrangement, more information can be found in Battisti et al. (2021).

Along the neighbouring coastal dunes, anthropogenic litter largely occur, mainly in autumn-winter period, after seastorms (Poeta et al. 2014; Gallitelli et al. 2021b). Inside wetland, anthropogenic litter is largely present, mainly the lighter polystyrene, moving from beaches, dune until inner wet areas (Battisti et al. 2020; Cresta and Battisti 2021).

Concerning coypu, in Torre Flavia wetland, this mammal has been largely studied locally (Marini et al. 2011; Battisti et al. 2015). Here, coypu was represented by a peripheral sink population (*sensu* Pulliam 1988) of a larger meta-population diffused on regional scale (i.e. large Tiber river valley), only occasionally interested by the occurrence of immigrant individuals that use this wet ecosystem as a temporary trophic and breeding site. These data showed as this rodent is here represented by a sink population, characterized by strong seasonal and interannual demographic oscillations (1.40–5.72/ind. 100 m), due to low connectivity among other surrounding groups. Locally, diet has been investigated, largely focused on rushbed plant species: 19 species, mainly hygrophilous monocotyledons and terrestrial dicotyledons, corresponding to about 25% of the available plants (*n* = 76 available plant species: 51 dicotyledons and 25 monocotyledons; Marini et al. 2013).

### Protocol and samples processing

We focused on field study on coypu’s faeces, recorded along a 100-m-long transect surrounding the Torre Flavia wetland. Overall, 30 faeces were sampled in situ and stored in containers. Then, in laboratory, faeces were controlled on their surface under stereoscope to discriminate that these tracks were contaminated, so that the MP in the tracks only came from inside them and not from outside. Thus, faeces were digested in 70 ml distilled water and 30 ml peroxide for a week at 20 °C (i.e. environmental room temperature, see methods in Nuelle et al. 2014 and Gallitelli et al. 2020). Then, digested faeces were analysed under stereoscope (see in Gallitelli et al. 2021a) for detecting possible microplastics. All the suspected found MP were isolated in a petri for being further confirmed as plastics. So, on a sample subset (10% of total suspected plastics), FT-IR analysis and technology was used to confirm the polymers.

To avoid plastic atmospheric contamination during analysis, nitrile gloves, clothes, and sterilised tweezers were used according to literature protocol (Gallitelli et al. 2020).

Due to licit operational limits, the characterization of anthropogenic particles was carried out only for items greater than 0.5 mm. In particular, isolated particles were on average smaller than 1 mm, and the characterization of microplastics extracted and isolated in a glass Petri dish was performed for particles > 0.5 mm and by Fourier Transform Infrared spectroscopy (FT-IR). In particular, the IR spectra were collected using Thermo Scientific Nicolette 6700 spectrophotometer, the spectrum range was 4000–400 cm^−1^ (100 scans, spectral resolution 4 cm^−1^). Chemical composition of polymer particles was identified by comparison with reference spectra database (instrument library and http://www.ftir-polymers.com/soon.htm). The identification was accepted when at least 80% of the peak frequencies corresponded in frequency of the reference spectrum.

### Statistical analysis

Before all analysis, data normality was confirmed using Shapiro–Wilk test. After that, parametric or non-parametric tests were used considering if distribution was normal or not normal. Results about faecal track weight and MP size were expressed as mean ± sd. To investigate if the number of anthropogenic particles is increasing with the faecal weight, we performed first a correlation and then a linear regression. These results were showed by two graphs, and residual plots were calculated for the linear regression. All statistical analyses were performed using GraphPad Prism 8.4.2.

## Results

**Fig. 2:**
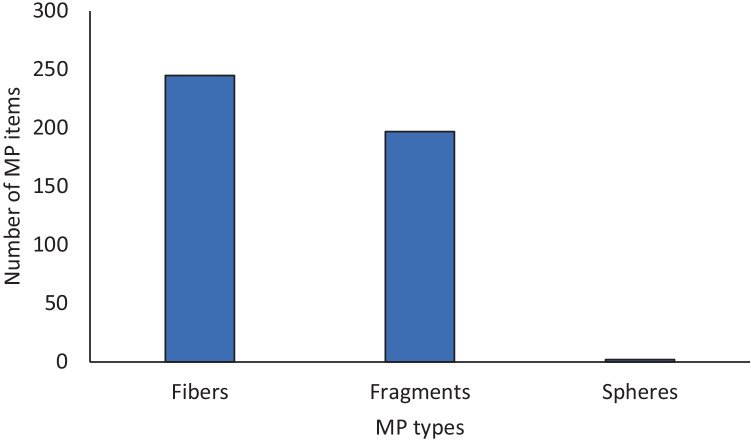
Anthropogenic litter occurring in coypu faeces are mainly fibres and fragments.

Totally, we recorded 30 faecal tracks with a medium weight of 4.58 ± 1.52 g. After analysis, excluding degraded vegetal remains, we recorded 444 natural and anthropogenic particles (Fig. 1). Specifically, most of items were fibers (*n* = 245, 55.2%), followed by fragments (*n* = 197, 44.4%), and spheres (*n* = 2, 0.5%; see Fig. 2).

Spectrophotometric analysis of the 10% of the particles recovered (> 0.5 mm) revealed that > 70% of them was not MP. Common non-plastic materials were hairs, sand, lignin, chitin, and other inorganic materials not further classified.

Among the polymers, we recorded polyethylene (PE), polyethylene terephthalate (PET), and polyamide (PA).

**Fig. 3:**
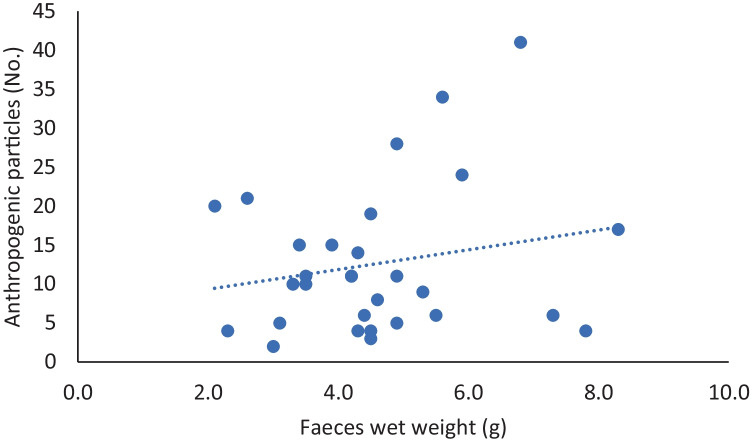
Not-significant correlation between number of faeces wet weight (g) and anthropogenic particles (No.)

**Fig. 4.**
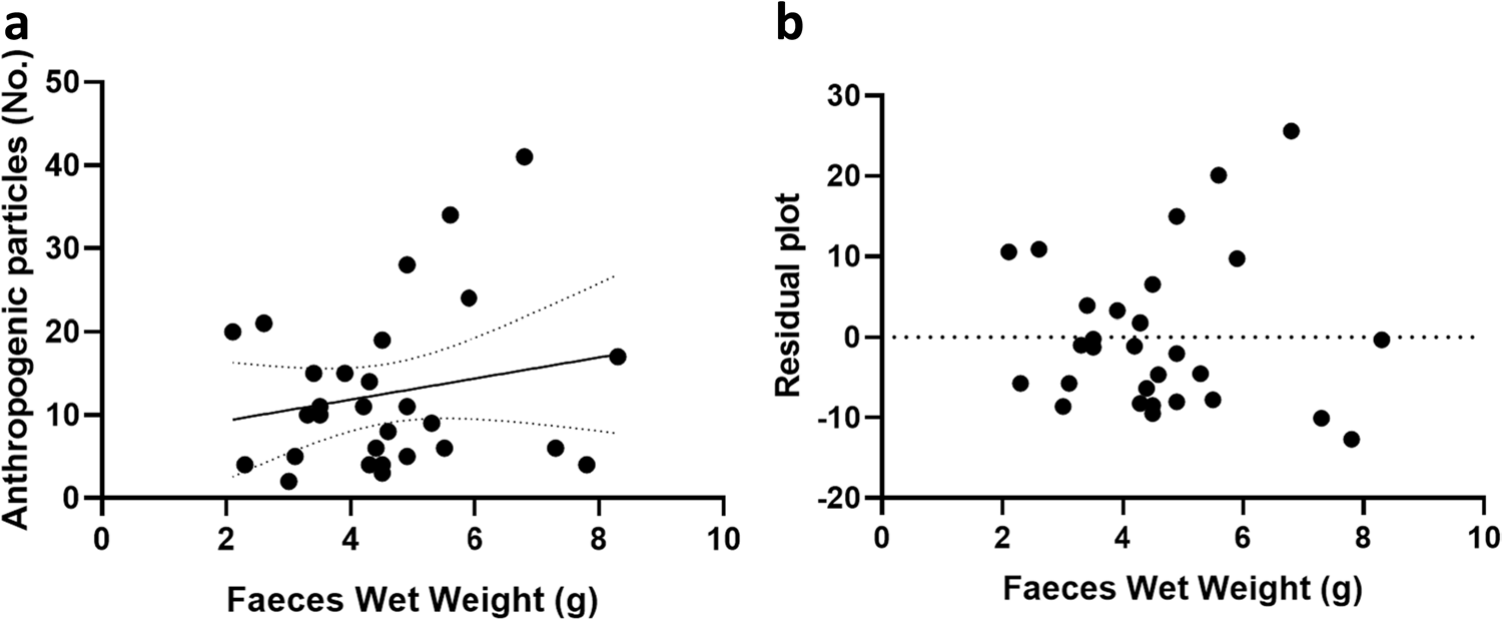
**a** Linear regression between faeces wet weight (g) and number (No.) of anthropogenic particles. **b** Residual plots for linear regression.

**Fig. 5:**
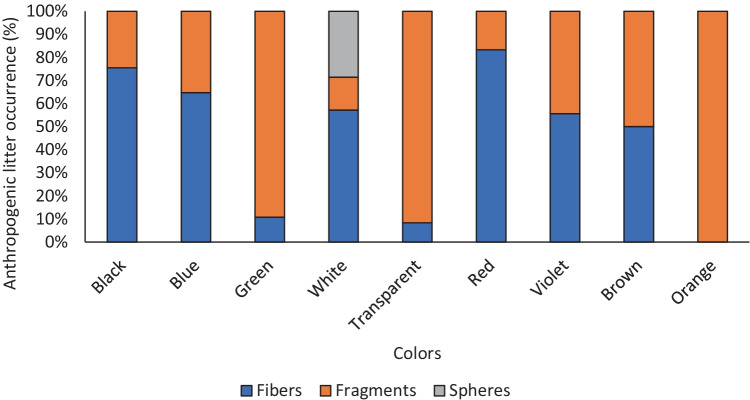
Most occurring colors in anthropogenic litter in coypu faeces.

The number of anthropogenic particles is not significantly correlated with the faecal weight (*r*_s_ = 0.13, *p* = 0.49, Fig. 3). Also, a linear regression resulted not significative (*r* = 0.04, *p* = 0.28, *Y* = 1.267*X + 6.795, Fig. 4). Standardised values of anthropogenic particles on track weight are reported in Table S1. Most found colours are blue (58.1%), followed by black (11.0%) and transparent (10.8%) (Fig. 5).

Among anthropogenic litter, the MP particles showed a size ranging between 0.25 and 5.90 mm, with a mean of 1.44 ± 1.08 mm and a total size of 63.26 mm (Table S2). In addition, one fibre resulted big as a mesoplastic item (length: 5.9 mm).

## Discussion

In this study, we report for the first time the presence of anthropogenic particles, including microplastics, in coypu faeces. The presence of these materials may be due to litter, involuntarily ingested with edible plants or present in the waters of the humid environments frequented by this species. The coypu occasionally feeds also on invertebrates (Wilsey et al. 1991), and the presence of anthropogenic litter cannot be excluded even in these preys which are then accumulated in the rodent. Moreover, as coypus can ingest their faeces being coprophagous (Gosling 1979), MP can turn back in their bodies - taking part into the trophic web. As many mammals may build their holts using anthropogenic plastic particles (see *Lutra lutra*: Kruuk and Kruuk 2006), also coypu could build plastic den as well. This might pose at risk this species due to plastic ingestion and entanglement (Azevedo-Santos et al. 2021). In general, although the amount of MP accumulated in coypu is unknown, and consequently MP may potentially have impacts on the animal health, future studies should be carried out to investigate more in detail this process.

**Fig. 6:**
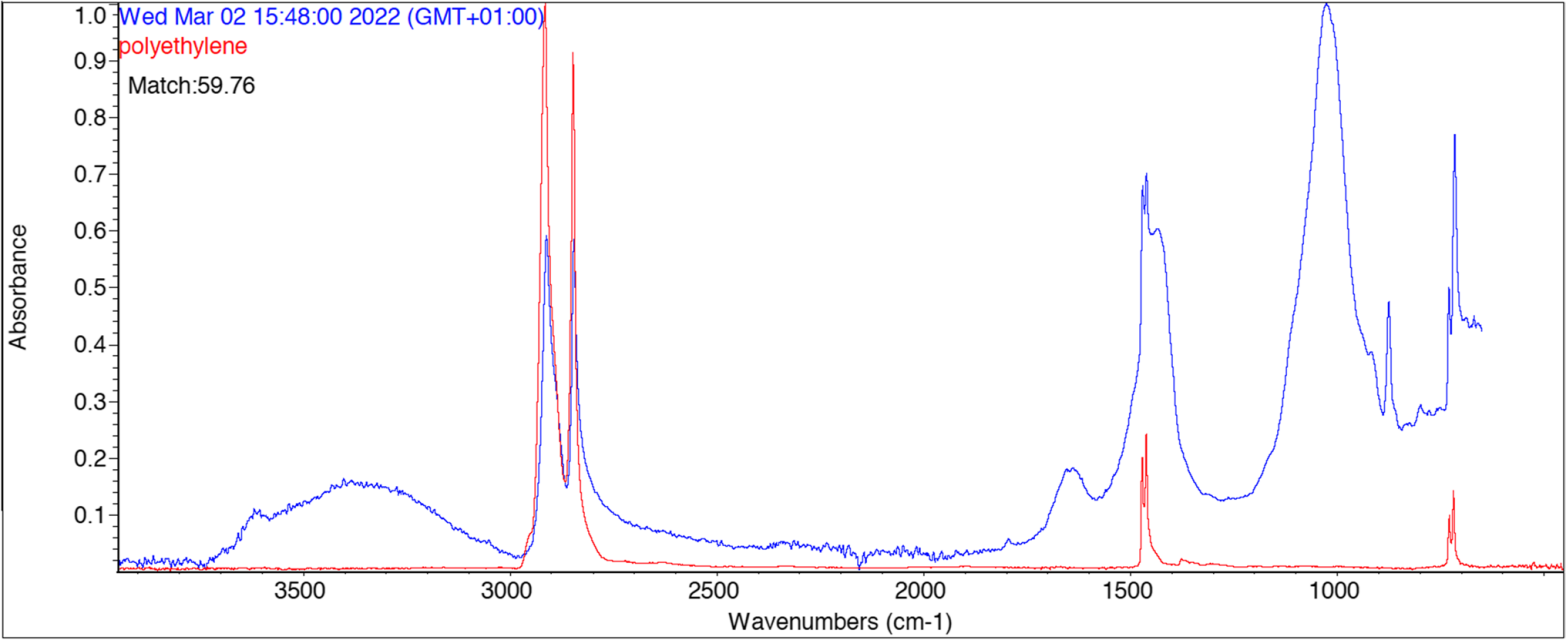
Degraded spectrum of polyethylene MP in coypu faeces.

Polyethylene (PE), polyethylene terephthalate (PET), and polyamide (PA) were the three most frequently polymers found. The presence of highly degraded MP could be due to prolonged action of the gastrointestinal tract of coypu in contact with digestive enzymes and gastric juices that could have an acid pH (Bano et al. 2017, for instance, see degraded polyethylene found in coypu faeces in Fig. 6). Concerning the plastic type, most of particles were fibres, and this could be due to this degradation process or even to a high environmental availability. According to recent studies, it can be speculated that plastic fragmentation is associated to mechanical forces and enzymatic processes (e.g. Mateos-Cárdenas et al. 2020). Also, O'Connor et al. (2022) reported that MP might be ingested and then egested by higher level organisms (the Eurasian otter *Lutra lutra* in Smiroldo et al. 2019) and that MP can rise through trophic web accumulating in predators due to biomagnification. However, MP in faeces may be originated not only by a feeding activity. Indeed, coypu could ingest MP because these particles could be located on the body, among the hairs. In literature, we found that MP can be carried by waterbird feathers (Reynolds and Ryan 2018).

Although MP along the food chain are poorly investigated, impact of fate of MP may play a central role in biodiversity and ecosystem health (Benson et al. 2022). Being at the top of food web, freshwater mammals could be important MP indicator, as well as dippers as they are food web apex (D’Souza et al. 2020). Furthermore, we should consider that a large number of particles was not categorized among MP, mainly due to the fact that particles are too small to be analysed with FT-IR procedure, so size particles showed a threshold of > 0.5 mm, while μ-FTIR would be needed to characterise smaller particles (i.e. < 0.5 mm). Indeed, the size of MP found in tracks would also justify the impossibility of characterising particles due to the lack of a μ-FTIR needed to characterise all the particles found. As the aim of the work is to verify the possibility of using coypu faeces as a bio-indicator, our effort represented a first step to verify the presence of MP in tracks, and therefore, it is sufficient to characterise those particles with a larger size. Operational limits in sampling procedure affected the range in particle size. This fact did not restrict the importance of our study since, independently from size, for the first time, we obtained evidence of plastic occurrence in this terrestrial/freshwater mammal.

A portion of this litter, which is difficult to quantify due to acid degradation from digestive processes, is made up of microplastics. Again, this is the first evidence of microplastics for this species. Although the presence of MP in marine mammals has been widely documented (review in Poeta et al. 2017; Santillán et al. 2020), these are the first evidence of these anthropogenic litter in a terrestrial/freshwater mammals. To our knowledge, the only other record of MP has been obtained for faeces in Eurasian otter (*Lutra lutra*; Smiroldo et al. 2019). In particular, they pointed out that two polymers were mainly identified as polyethylene terephthalate (PET) and polyamide (PA). Thus, our result is in line with literature. In our case, the presence of MP could be due to the local high environmental availability of MP occurring in this coastal wetland as discussed also in Gallitelli et al. (2021b) and Cresta and Battisti et al. (2021).

Differently from otter, a threatened localized species, coypu (both in native and non-native ranges) may be used as bioindicator of MP pollution, due to their widespread and wide distribution. Indeed, this species meets many of the typical criteria requested for a biological indicator (Noss 1990): (i) coypu has a stable zoological systematic and taxonomy; (ii) it is a relatively generalist, common, widespread, and medium-sized species, relatively easy to detect and to sample tracks with a minimum field and economic effort; (iii) it is sensitive to collect a specific variable (in our case, anthropogenic particles and, among them, microplastics); (iv) it is universally applicable and spatially comparable: this largely diffused species show a similar ecology inside their geographical range (Reeves and Usher 1989); and (v) interpretation and communication of results may be easily carried out through simple, quantitative, and reproducible variables (e.g. number/density of MP).

Analogous to coypu, other alien (i.e. non-native) species have been used in this regard for plastic pollution detection: for example, in Australian wetlands the common mosquito fish (*Gambusia holbrooki*) has been used for monitoring MP (Su et al. 2019).

In conclusion, evidence on plastics in faeces are totally scarce for terrestrial species (both wild and domestic species, Beriot et al. 2021). Here, our results addressed that coypu ingest plastics and egest them through faeces. Also, coypu seems to be a species able to provide a proxy of environmental bioavailability of microplastics. However, the data we presented are of exceptional nature, data that for the first time recorded the presence of anthropogenic plastic material in the faeces of a terrestrial mammal outside its original range of distribution. Further research is necessary to (i) have more consideration on the new produced plastics (i.e. the relation to the size of the ingested plastics by animals, Jâms et al. 2020) and (ii) to define standardized protocols useful to obtain comparable information using MP as indicator in this common freshwater mammal. More in detail, to develop a biomonitoring plan based on the analysis of faecal tracks, there is the need of understanding if the real concentration of MP pollution can be given by faecal tracks or if there are many losses of MP along the ingestion, digestion, and egestion processes by coypu. All these future studies would have a central role as these new degraded plastics (i.e. small microplastics and nanoplastics) might have a potential impact to animal health and consequently to ecosystem functioning.

## Supplementary information

ESM 1 (DOCX 20 kb)

## Data Availability

Not applicable
