# Supplementary material for: Anthropogenic particles in coypu (Myocastor coypus; Mammalia, Rodentia)’ faeces: first evidence and considerations about their use as track for detecting microplastic pollution
Source: Environ Sci Pollut Res Int. 2022 Jun 4;29(36):55293–301. doi: 10.1007/s11356-022-21032-0 (PMC9356950; doi:10.1007/s11356-022-21032-0)
Supplement: Supplementary file 1 — (DOCX 20 kb) [file 11356_2022_21032_MOESM1_ESM.docx]

**Supplementary Materials**

Anthropogenic particles in coypu (*Myocastor coypus*; Mammalia, Rodentia)’ faeces: first evidence and considerations about their use as track for detecting microplastic pollution

Luca Gallitelli^1*^, Corrado Battisti^2^, Loris Pietrelli^3^, Massimiliano Scalici^1^

^1^ Department of Sciences, University of Rome Tre, Rome, Italy*, ORCID:* [*0000-0002-2188-4584*](mailto:0000-0002-2188-4584)*; Massimiliano Scalici, ORCID:* [*0000-0002-5677-8837*](https://orcid.org/0000-0002-5677-8837)*.*

^2^ ’Torre Flavia’ LTER (Long Term Ecological Research) Station, Protected Areas Service,

Città Metropolitana di Roma Capitale, Rome, Italy; ORCID: 000-0002-2621-3659

^3^ Department of Chemistry, Sapienza University of Rome, P.le A. Moro, 5, 00185, Rome, Italy, ORCID: 0000-0002-3825-1468

^*^corresponding author: [*luca.gallitelli@uniroma3.it*](mailto:luca.gallitelli@uniroma3.it)

**Table S1**. Number of anthropogenic particles (No.) in track samples, with reporting wet weight (g) and standardised values of MP items on faecal weight.

| **Samples** | **MP (No.)** | **Wet Weight (g)** | **MP items (No.)/ track weight (g)** |
| --- | --- | --- | --- |
| 1 | 19 | 4.5 | 4.222222222 |
| 2 | 6 | 7.3 | 0.821917808 |
| 3 | 17 | 8.3 | 2.048192771 |
| 4 | 11 | 4.2 | 2.619047619 |
| 5 | 11 | 4.9 | 2.244897959 |
| 6 | 10 | 3.3 | 3.03030303 |
| 7 | 41 | 6.8 | 6.029411765 |
| 8 | 9 | 5.3 | 1.698113208 |
| 9 | 34 | 5.6 | 6.071428571 |
| 10 | 6 | 4.4 | 1.363636364 |
| 11 | 4 | 4.3 | 0.930232558 |
| 12 | 11 | 4.2 | 2.619047619 |
| 13 | 6 | 5.5 | 1.090909091 |
| 14 | 5 | 4.9 | 1.020408163 |
| 15 | 3 | 4.5 | 0.666666667 |
| 16 | 28 | 4.9 | 5.714285714 |
| 17 | 14 | 4.3 | 3.255813953 |
| 18 | 4 | 7.8 | 0.512820513 |
| 19 | 24 | 5.9 | 4.06779661 |
| 20 | 8 | 4.6 | 1.739130435 |
| 21 | 2 | 3 | 0.666666667 |
| 22 | 10 | 3.5 | 2.857142857 |
| 23 | 4 | 4.5 | 0.888888889 |
| 24 | 4 | 2.3 | 1.739130435 |
| 25 | 20 | 2.1 | 9.523809524 |
| 26 | 21 | 2.6 | 8.076923077 |
| 27 | 15 | 3.4 | 4.411764706 |
| 28 | 5 | 3.1 | 1.612903226 |
| 29 | 15 | 3.9 | 3.846153846 |
| 30 | 11 | 3.5 | 3.142857143 |

**Table S2**. Size of recognised MP (mm) found within faecal tracks.

| **MP (No.)** | **MP size (mm)** |
| --- | --- |
| 1 | 1 |
| 2 | 1.25 |
| 3 | 5.9 |
| 4 | 4 |
| 5 | 2 |
| 6 | 2.5 |
| 7 | 1 |
| 8 | 3 |
| 9 | 1.5 |
| 10 | 0.5 |
| 11 | 1.1 |
| 12 | 0.55 |
| 13 | 1 |
| 14 | 2.5 |
| 15 | 0.5 |
| 16 | 0.5 |
| 17 | 1.5 |
| 18 | 2.1 |
| 19 | 2.6 |
| 20 | 0.5 |
| 21 | 1 |
| 22 | 0.6 |
| 23 | 1 |
| 24 | 1.5 |
| 25 | 0.55 |
| 26 | 0.45 |
| 27 | 1.5 |
| 28 | 0.5 |
| 29 | 2.5 |
| 30 | 1 |
| 31 | 2 |
| 32 | 1.5 |
| 33 | 0.5 |
| 34 | 0.25 |
| 35 | 1.25 |
| 36 | 0.5 |
| 37 | 1.5 |
| 38 | 0.45 |
| 39 | 2.4 |
| 40 | 1 |
| 41 | 0.56 |
| 42 | 1.5 |
| 43 | 1.55 |
| 44 | 2.2 |
